# Supplementary material for: From sequence to enzyme mechanism using multi-label machine learning
Source: BMC Bioinformatics. 2014 May 19;15:150. doi: 10.1186/1471-2105-15-150 (PMC4229970; doi:10.1186/1471-2105-15-150)
Supplement: Additional file 2 — Java code of ml2db. Additional file ml2db_code.tar.gz contains the Java source code to run the multi-label machine learning experiments and save the results to database. The code’s Javadoc is included. [file 1471-2105-15-150-S2.zip › additional file 2/ml2db/ml2db/doc/mulan/classifier/meta/package-tree.html]

mulan.classifier.meta Class Hierarchy


JavaScript is disabled on your browser.


- Overview
- Package
- Class
- Use
- Tree
- Deprecated
- Index
- Help

- Prev
- Next

- Frames
- No Frames

- All Classes

# Hierarchy For Package mulan.classifier.meta

Package Hierarchies:

- All Packages

## Class Hierarchy

- java.lang.Object
  - weka.clusterers.AbstractClusterer (implements weka.core.CapabilitiesHandler, java.lang.Cloneable, weka.clusterers.Clusterer, weka.core.RevisionHandler, java.io.Serializable)
    - weka.clusterers.RandomizableClusterer (implements weka.core.OptionHandler, weka.core.Randomizable)
      - mulan.classifier.meta.ConstrainedKMeans (implements weka.clusterers.NumberOfClustersRequestable, weka.core.WeightedInstancesHandler)
  - mulan.classifier.meta.ConstrainedKMeans.bucketInstance (implements java.lang.Comparable<T>)
  - mulan.classifier.meta.HierarchyBuilder (implements java.io.Serializable)
  - mulan.classifier.MultiLabelLearnerBase (implements mulan.classifier.MultiLabelLearner, java.io.Serializable, weka.core.TechnicalInformationHandler)
    - mulan.classifier.meta.MultiLabelMetaLearner
      - mulan.classifier.meta.ClusteringBased
      - mulan.classifier.meta.EnsembleOfSubsetLearners
      - mulan.classifier.meta.HMC
      - mulan.classifier.meta.HMCNode
      - mulan.classifier.meta.HOMER
      - mulan.classifier.meta.RAkEL
      - mulan.classifier.meta.RAkELd
      - mulan.classifier.meta.SubsetLearner

## Enum Hierarchy

- java.lang.Object
  - java.lang.Enum<E> (implements java.lang.Comparable<T>, java.io.Serializable)
    - mulan.classifier.meta.HierarchyBuilder.Method

- Overview
- Package
- Class
- Use
- Tree
- Deprecated
- Index
- Help

- Prev
- Next

- Frames
- No Frames

- All Classes
